# Postoperative pneumonia following cardiac surgery in non-ventilated patients versus mechanically ventilated patients: is there any difference?

**DOI:** 10.1186/s13054-015-0845-5

**Published:** 2015-03-11

**Authors:** Nicolas Allou, Jerome Allyn, Aurélie Snauwaert, Camille Welsch, Jean Christophe Lucet, Rita Kortbaoui, Mathieu Desmard, Pascal Augustin, Philippe Montravers

**Affiliations:** Hôpital Universitaire Félix Guyon, Réanimation Polyvalente, Allée des Topazes, 97400 Saint Denis, France; AP-HP, Hôpital Bichat, Département d’Anesthésie-Réanimation, 46 rue Henri Huchard, F-75018 Paris, France; Univ Paris Diderot, Sorbonne Paris Cité, F-75018 Paris, France; AP-HP, Hôpital Bichat, Unité d’Hygiène et de lutte Contre les Infections Hospitalières, 46 rue Henri Huchard, F-75018 Paris, France

## Abstract

**Introduction:**

No studies have compared ventilator-associated pneumonia (VAP) and non-VAP following cardiac surgery (CS). The aim of this study was to assess the incidence, clinical and microbiologic features, treatment characteristics and prognosis of postoperative pneumonia following CS with a special focus on non-VAP.

**Methods:**

This was a retrospective cohort study based on a prospectively collected database. We compared cases of non-VAP and VAP following CS observed between January 2005 and December 2012. Statistical analysis consisted of bivariate and multivariate analysis.

**Results:**

A total of 257 (3.5%) of 7,439 consecutive CS patients developed postoperative pneumonia, including 120 (47%) cases of non-VAP. Patients with VAP had more frequent history of congestive heart failure (31% vs. 17%, *P* = 0.006) and longer duration of cardiopulmonary bypass (105 vs 76 min, *P* < 0.0001), than patients with non-VAP. No significant differences were observed between the 2 groups in terms of the types of microorganisms isolated with high proportions of Enterobacteriaceae (35%), *Pseudomonas aeruginosa* (20.2%) and *Haemophilus spp* (20.2%), except for a lower proportion of Methicillin-susceptible *S. aureus* in the non-VAP group (3.2% vs 7.9%, *P* = 0.03). In the intensive care unit, patients with non-VAP had lower sequential organ failure assessment scores than patients with VAP (8 ± 3 versus 9 ± 3, *P* = 0.004). On multivariate analysis, in-hospital mortality was similar in both groups (32% in the non-VAP group and 42% in the VAP group, adjusted Odds Ratio (aOR): 1.4; 95% confidence intervals (CI): 0.7-2.5; *P* = 0.34) and appropriate empiric antibiotic therapy was associated with a reduction of in-hospital mortality (aOR: 0.4; 95% CI: 0.2-1; *P* = 0.05). Piperacillin/tazobactam or imipenem monotherapy constituted appropriate empiric therapy in the two groups, with values reaching 93% and 95% with no differences between VAP and non-VAP cases.

**Conclusions:**

Intensive care patients with VAP are more severely ill than non-VAP patients following CS. Nevertheless, patients with non-VAP and VAP following CS have similar outcomes. This study suggests that the empiric antibiotic regimen in patients with pneumonia following CS should include at least a broad-spectrum antibiotic targeting non-fermenting Gram-negative bacilli, regardless of the type of pneumonia, and targeting *S. aureus* in VAP patients.

**Electronic supplementary material:**

The online version of this article (doi:10.1186/s13054-015-0845-5) contains supplementary material, which is available to authorized users.

## Introduction

Postoperative pneumonia (POP), is the most common infection following cardiac surgery (CS) [1,2] and constitutes a major source of morbidity and mortality [1,2]. Previous studies confined their analysis to patients with ventilator-associated pneumonia (VAP) [1-4]. However, VAP is not the only presentation of postoperative pneumonia (POP) following surgery in general (36%) [5] and CS in particular (57%) [6]. Extrapolation of data on VAP following CS to non-VAP may, therefore, lead to significant errors in the management of patients with non-VAP. The aim of this study was to compare the characteristics of VAP and non-VAP following CS. In order to define precisely the clinical characteristics and antibiotic therapy of these patients, we assessed the incidence, clinical and microbiologic features, treatment characteristics and prognosis of a group of patients who developed non-VAP following CS.

## Materials and methods

Data from consecutive patients who underwent CS with cardiopulmonary bypass from January 2005 to December 2012 in a 1,200-bed university hospital were included in this single-center cohort study. Perioperative characteristics were obtained from our local, prospectively collected database. The study was approved by the local ethics committee, which waived the need for informed consent, because of the observational nature of the study (Institutional Review Board 00006477, Paris 7 University, AP-HP). Patients on mechanical ventilation before CS were excluded from the analysis.

Perioperative care, including anesthesia, monitoring techniques and normothermic cardiopulmonary bypass, was standardized for all patients. During the study period, patients received a systematic decolonization protocol with three doses of intranasal mupirocin per day for 5 consecutive days. Cefamandole (30 mg/kg intravenously one hour before sternotomy, 750 mg (1,500 mg in patients with body mass index >35 kg/m^2^) every two hours during surgery and continued for 24 hours in the ICU at a dosage of 750 mg, four times per day) was used for surgical antibiotic prophylaxis. In patients with beta-lactam allergy, antibiotic prophylaxis comprised vancomycin (15 mg/kg intravenously over 60 minutes one hour before sternotomy and 15 mg/kg at the eighth hour) + gentamicin (3 mg/kg intravenously as a single dose). No subsequent prophylaxis was administered. No subglottic aspiration system was used. After surgery patients were transferred to the ICU without systematic postoperative sedation and were extubated when the standard extubation criteria were obtained (hemodynamically stable; no significant bleeding and percutaneous oxygen saturation >95% with inspired oxygen fraction (FiO_2_) <0.4).

Prevention of postoperative pneumonia included orotracheal intubation, cleaning of the oropharyngeal cavity with 0.1% hexetidine solution (McNeil, Issy-les-Moulineaux, France) four times per day, semirecumbent body positioning (30 to 45°), tracheal cuff pressure maintained between 20 and 30 cm H_2_O, and physiotherapy (at least twice daily for 2 days following extubation).

### Diagnosis of postoperative pneumonia

Postoperative pneumonia was suspected on the basis of a new lung infiltrate on daily chest radiographs associated with at least two of the following findings: temperature ≥38.3°C or <36°C, white blood cell count >12,000 cells/mm^3^ or <5,000 cells/mm^3^, and purulent secretion.

Postoperative pneumonia was systematically confirmed by microbiologic analysis using bronchoalveolar lavage with fiberoptic bronchoscopy (yielding bacteria at a concentration of >10^4^ colony forming units (CFU)/mL) or blind protected distal bronchial specimen samples (yielding >10^3^ CFU/mL) or blind endotracheal aspiration (yielding >10^5^ CFU/mL). Only the first episode of pneumonia following surgery was studied and was defined as POP. VAP was defined as pneumonia occurring in patients with postoperative invasive mechanical ventilation for 48 hours or longer with the ventilator in place at the time of or 24 hours before the event [7]. Patients who developed POP but who did not meet the criteria for VAP were defined as non-VAP. Early-onset POP was defined as pneumonia with onset <5 days following surgery and late-onset POP was defined as pneumonia with onset ≥5 days following surgery [8].

### Susceptibility testing and empiric antimicrobial therapy

Empiric antimicrobial therapy based on local guidelines was always initiated in the case of suspected POP and was considered appropriate when all bacteria isolated at a significant concentration were susceptible to at least one of the drugs administered, according to susceptibility testing. The choice of antibiotic therapy was left to the attending physician and was guided by Gram-stained direct examination and the severity of pneumonia at the time of diagnosis. Susceptibility testing was determined by the disk-diffusion method, according to the criteria of the Antibiogram Committee of the French Society for Microbiology [9].

### Data collected

Demographic characteristics and underlying diseases were recorded during the first 24 hours after admission to the ICU. Intraoperative and postoperative characteristics and outcome (ICU length of stay and in-hospital mortality) were also recorded.

### Statistical analysis

Results were expressed as total numbers (percentage) for categorical variables and mean ± standard deviation or median (25^th^ to 75^th^ percentiles) as appropriate for continuous variables. Continuous variables were compared by the unpaired Student *t*-test or nonparametric (Mann-Whitney) test as appropriate. Categorical variables were compared by the chi-square test or Fisher’s exact test as appropriate. Risk factors found to be predictive of postoperative mortality in bivariate analysis *P*-values <0.1 were entered into a multivariate logistic regression analysis using backward selection on a criterion of *P* <0.05. A *P*-value <0.05 was considered significant. Calibration was assessed using the Hosmer and Lemeshow goodness-of-fit test. Analysis was performed using SAS statistical software (8.2, Cary, NC, USA).

## Results

### Study population

From January 2005 to December 2012, 7,468 cardiac procedures with cardiopulmonary bypass were performed. Twenty-nine patients were on mechanical ventilation before surgery and were excluded from the analysis. The remaining 7,439 patients constituted the cohort. Overall, 257 (3.5%; 95% CI 3.1, 3.9) of 7,439 consecutive CS patients developed POP including 120 (46.7%) cases of non-VAP. POP was diagnosed 4.3 ± 2 days after CS in both groups (*P* = 0.55). Ninety patients (66%) developed early-onset POP in the VAP group versus 76 patients (63%) in the non-VAP group (*P* = 0.69). Preoperative and intraoperative characteristics of the 257 patients are presented in Tables 1 and 2.

**Table 1:** **Preoperative characteristics**

**Variable**	**Patients with**	**Patients with**	***P-*** **value**
	**VAP (n =137)**	**non-VAP (n =120)**	
EuroSCORE, %, mean ± SD	10.7 ± 9.9	10.2 ± 12.9	0.70
Age, yr, mean ± SD, ()	68.2 ± 11.2	69.6 ± 13.0	0.34
Male gender, n (%)	98 (71.5)	86 (71.7)	0.78
Body mass index, kg/m^2^, mean ± SD	26.7 ± 4.6	26.1 ± 4.7	0.32
Comorbidities			
Cancer, n (%)	5 (3.6)	3 (2.5)	0.60
Mediastinal irradiation, n (%)	4 (2.9)	6 (5)	0.39
Liver cirrhosis, n (%)	1 (0.7)	4 (3.3)	0.29
Decompensated liver cirrhosis, n (%)	1 (0.7)	1 (0.8)	0.99
Dyslipidemia, n (%)	62 (45.3)	55 (45.8)	0.93
COPD, n (%)	29 (21.2)	34 (28.3)	0.18
COPD with treatment, n (%)	21 (15.3)	24 (20)	0.33
Hypertension, n (%)	81 (59.1)	78 (65)	0.33
Immunosuppression, n (%)^a^	1 (0.7)	3 (2.5)	0.52
History of coronary artery disease, n (%)	66 (48.2)	65 (54.2)	0.34
History of congestive heart failure, n (%)	43 (31.4)	20 (16.7)	0.006
Neurologic disease, n (%)^b^	6 (4.4)	4 (3.3)	0.67
History of stroke, n (%)	17 (12.4)	16 (13.3)	0.83
Peripheral vascular disease, n (%)	23 (16.8)	27 (22.5)	0.25
Diabetes mellitus, n (%)	41 (29.9)	38 (31.7)	0.76
History of smoking, n (%)	67 (48.9)	70 (58.3)	0.13
Current smoking, n (%)^c^	14 (10.2)	27 (22.5)	0.007
Creatinine clearance, mL/min, mean ± SD	63.9 ± 26.5	65.7 ± 33.7	0.62
Chronic renal failure with dialysis, n (%)	1 (0.7)	1 (0.8)	0.99
Atrial fibrillation, n (%)	60 (43.8)	36 (30)	0.02
New York Heart Association class ≥ III, n (%)	107 (78.1)	80 (66.7)	0.04
Left ventricular ejection fraction, %, mean ± SD	53 ± 15	55 ± 12	0.18
Prior antibiotic therapy, n (%)	29 (21.2)	25 (20.8)	0.95
Preoperative treatment, n (%)			
Angiotensin-converting enzyme inhibitor	66 (48.2)	62 (51.7)	0.58
Betablocker	73 (53.3)	58 (48.3)	0.43
Antiplatelet therapy	80 (58.4)	76 (63.3)	0.42
Statin	74 (54)	76 (63.3)	0.13

^a^Including any immunosuppressive diseases, hematologic diseases, treatment with immunosuppressive drugs within the previous 30 days, or corticosteroids at daily doses of at least 10 mg/day of a prednisone equivalent for more than 2 weeks. ^b^Neurologic disease associated with functional disability. ^c^Subject smoked an average of at least five cigarettes per day during the month before surgery. COPD, chronic obstructive pulmonary disease; VAP, ventilator-associated pneumonia.

**Table 2:** **Intraoperative characteristics**

**Variable**	**Patients with**	**Patients with**	***P-*** **value**
	**VAP (n =137)**	**non-VAP (n =120)**	
Non-scheduled surgery, n (%)	13 (9.5)	6 (5)	0.17
Redo surgery, n (%)^a^	22 (16.1)	12 (10)	0.15
Coronary artery bypass grafting, n (%)	61 (44.5)	64 (53.3)	0.16
Valvular surgery, n (%)	90 (65.7)	65 (54.2)	0.06
Systolic PAP, mmHg, mean ± SD	37 ± 14	35 ± 12	0.36
Congestive heart failure, n (%)	19 (13.9)	9 (7.5)	0.1
Acute renal insufficiency, n (%)	4 (2.9)	1 (0.8)	0.23
Venoarterial ECMO at the end of surgery, n (%)	5 (3.6)	2 (1.7)	0.33
Antibiotic prophylaxis with cefamandole, n (%)	115 (83.9)	104 (86.7)	0.54
Difficult endotracheal intubation, n (%)	6 (4.4)	8 (6.7)	0.42
Transesophageal echocardiography, n (%)	72 (52.6)	38 (32.7)	0.0007
Cardiopulmonary bypass time, minutes, mean ± SD	105 ± 63	76 ± 41	<0.0001
Aortic clamp time, minutes, mean ± S	69 ± 35	57 ± 29	0.002
Operating time, minutes, mean ± S	237 ± 84	200 ± 56	<0.0001
Inotropic support at the end of surgery, n (%)	88 (64.2)	50 (41.7)	0.0003
Intraoperative number of units of PRBC, n (%)	1.8 ± 2.1	1.2 ± 1.6	0.03

^a^Cardiac surgery requiring resternotomy. ECMO, extracorporeal membrane oxygenation; PAP, pulmonary artery pressure; PRBC, packed red blood cells; VAP, ventilator-associated pneumonia.

### Diagnostic procedures and distribution of microorganisms

Two hundred and sixty-eight respiratory samples were obtained from these 257 patients. Eleven patients had a first negative sample (7 protected distal bronchial specimen samples, 4 endotracheal aspirations) followed by a Preoperative characteristicssecond positive sample (11 bronchoalveolar lavage samples); 8 out of 120 (6.7%) patients in the non-VAP group versus 3 out of 137 (2.2%) patients in the VAP group (*P* = 0.08). Microbiologic samples were obtained with a bronchoscopic method (bronchoalveolar lavage) in 87 patients (63.5%) in the VAP group versus 72 patients (60%) in the non-VAP group (*P* = 0.56).

A total of 302 microorganisms were cultured at significant concentrations (Table 3). Postoperative pneumonia was polymicrobial in 101 (39.3%) cases with no significant differences between the two groups of patients (*P* = 0.29). The microorganisms most commonly isolated were Enterobacteriaceae (35.4%), *Pseudomonas aeruginosa* (20.2%) and *Haemophilus spp* (20.2%).

**Table 3:** **Microorganisms isolated from 257 episodes of postoperative pneumonia**

**Microorganisms, n (%)**	**VAP**	**Non-VAP**	***P-*** **value**
	**(n =137)**	**(n =120)**	
Polymicrobial infection	58 (42.3)	43 (35.8)	0.29
Non-fermenting Gram-negative bacilli	40 (29.2)	27 (22.5)	0.22
*Pseudomonas aeruginosa*	35 (25.5)	26 (21.7)	0.47
*Acinetobacter baumannii*	3 (2.2)	0	0.29
*Stenotrophomonas maltophilia*	2 (1.5)	1 (0.8)	0.99
Enterobacteriaceae	60 (43.8)	47 (39.2)	0.53
*Escherichia coli*	15 (10.9)	13 (10.8)	0.98
*Enterobacter spp*	14 (10.2)	6 (5)	0.12
*Klebsiella spp*	8 (5.8)	11 (9.2)	0.31
*Hafnia alvei*	4 (2.9)	4 (3.3)	0.85
*Citrobacter spp*	6 (4.4)	5 (4.2)	0.93
*Proteus spp*	7 (5.1)	6 (5)	0.97
*Morganella morganii*	1 (0.7)	0	0.99
*Serratia marcescens*	5 (3.6)	2 (1.7)	0.33
*Staphylococcus aureus*			
Methicillin-susceptible *S. aureus*	14 (10.2)	4 (3.3)	0.031
Methicillin-resistant *S. aureus*	1 (0.7)	0	0.99
*Haemophilus spp*	34 (24.8)	27 (22.5)	0.66
*Neisseria spp*	9 (6.6)	6 (5)	0.59
*Streptococcus spp*	20 (14.6)	13 (10.8)	0.37
*Streptococcus pneumoniae*	2 (1.5)	7 (5.8)	0.057
Other streptococci	18 (13.1)	6 (5)	0.025
Total microorganisms isolated	178	124	

VAP, ventilator-associated pneumonia.

No significant differences were observed between the two groups in terms of the relative proportion of microorganisms isolated except for a lower proportion of Methicillin-susceptible *S. aureus* in the non-VAP group (3.2% versus 7.9%, *P* = 0.03). No significant differences were observed between early-onset and late-onset POP in terms of the microorganisms isolated (*P* > 0.2 for all isolated microorganisms).

### Empiric antibiotic therapy and susceptibility of microorganisms

Two hundred and four patients had a dual combination with broad-spectrum antibiotic agents targeting non-fermenting Gram-negative bacilli (79.4%). The most commonly prescribed empiric antibiotic regimen was a combination of piperacillin/tazobactam and amikacin (70.4%) with no significant difference between the two groups (*P* = 0.89). A similar rate of appropriate empiric antimicrobial therapy was observed in the two groups (87.5% in non-VAP and 89.8% in VAP, *P* = 0.6). When empiric antibiotic therapy was inappropriate, the final antibiotic therapy was modified to achieve appropriateness in every case (2 days after the respiratory sample). Most microorganisms isolated in this study cohort were susceptible to antibiotic therapy including a broad-spectrum antibiotic agent targeting non-fermenting Gram-negative bacilli (>92%) with no significant differences between the two groups of patients (Table 4).

**Table 4:** **Susceptibility to different antimicrobial regimens of the strains responsible for postoperative pneumonia**

**Antibiotics, n (%)**	**VAP (n = 137)**	**Non-VAP (n = 120)**	***P-*** **value**
Monotherapy			
Amoxicillin/clavulanic acid	69 (50.4)	69 (57.5)	0.38
3GC with limited broad spectrum	96 (70.1)	91 (75.8)	0.3
Piperacillin/tazobactam	127 (92.8)	114 (95.0)	0.45
Imipenem	131 (95.6)	115 (95.8)	0.93
Dual combination			
Amoxicillin/ clavulanic acid/gentamicin	99 (72.3)	93 (77.5)	0.34
Piperacillin/tazobactam/amikacin	134 (97.8)	118 (98.3)	0.76
Piperacillin/tazobactam/gentamicin	129 (94.2)	114 (95.0)	0.77
Imipenem/Amikacin	134 (97.8)	118 (98.3)	0.76

VAP, ventilator-associated pneumonia; 3GC, 3rd generation cephalosporins.

### Outcome

Clinical characteristics of postoperative pneumonia at the time of diagnosis in the 257 patients are presented in Table 5.

**Table 5:** **Clinical characteristics of postoperative pneumonia at the time of diagnosis in the 257 patients**

**Variable**	**Patients with VAP (n = 137)**	**Patients with non-VAP (n = 120)**	***P-value***
CPIS score, mean ± SD	7.1 ± 1.2	6.9 ± 1.3	0.16
SOFA score, mean ± SD	9.1 ± 2.8	8.0 ± 3.1	0.004
Time to onset of pneumonia, days, mean ± SD	4 ± 2	4 ± 2	0.55
Peak value of cTnI, ng/mL, mean ± SD	18.7 ± 36.3	7.9 ± 11.5	0.002
Inotropic support with dobutamine, n (%)	103 (75.2)	69 (57.5)	0.003
Inotropic support with norepinephrine, n (%)	95 (69.3)	80 (66.7)	0.65
Inhaled nitrous oxide, n (%)	34 (24.8)	16 (13.3)	0.02
Number of units of PRBC, mean ± SD	2.2 ± 3.0	1.3 ± 1.9	0.005

CPIS, clinical pulmonary infection score; cTnI, cardiac troponin I; PRBC, packed red blood cells; SOFA, sequential organ failure assessment; VAP, ventilator-associated pneumonia.

Among the 120 patients with non-VAP, 93 (78%) required re-intubation during the 24 hours following the diagnosis of POP. Of the 257 patients, 95 (37%) died after heart surgery, including 38 (32%) of the 120 patients with non-VAP versus 57 (42%) of the 137 patients with VAP (*P* = 0.1). Of the 29 patients who received inappropriate empiric antibiotic therapy, 18 (62%) died versus 77 (34%) of the 228 patients with appropriate initial empiric therapy (*P* = 0.003).

### Risk factors for in-hospital mortality

Fifteen variables had a *P-*value < 0.10 on univariate analysis (see Additional file 1 for univariate analysis of risk factors for in-hospital mortality with *P* <0.10) and were entered into the logistic regression (Table 6).

**Table 6:** **Risk factors independently associated with in-hospital mortality in the 257 patients assessed on multivariate analysis**

**Variables**	**Adjusted odds ratio (95% CI)**	***P-*** **value**
History of congestive heart failure	2.28 (1.17, 4.46)	0.02
Non-fermenting Gram-negative bacilli	2.28 (1.18, 4.40)	0.01
Age	1.03 (1.01, 1.06)	0.02
Time to onset of postoperative pneumonia	1.18 (1.01, 1.39)	0.04
Diabetes mellitus	2.26 (1.19, 4.30)	0.01
Appropriate antibiotic therapy	0.40 (0.16, 1.00)	0.05
Angiotensin-converting enzyme inhibitor	0.41 (0.23, 0.74)	0.003
Antibiotic prophylaxis with Cefamandole	0.46 (0.19, 0.93)	0.03

The Hosmer-Lemeshow goodness-of-fit test showed good calibration of the model (*P* = 0.87). The Nagelkerke and Cox/Snell *R*-squares were 0.29 and 0.22, respectively.

On multivariate analysis (Table 6), in-hospital mortality was independently associated with a history of congestive heart failure (odds ratio (OR) 2.28, 95% CI 1.17, 4.46, *P* = 0.016), postoperative pneumonia due to non-fermenting Gram-negative bacilli (OR 2.28, 95% CI 1.18, 4.40; *P* = 0.01), age (OR 1.03, 95% CI 1.01, 1.06; *P* = 0.02), time to onset of POP (OR 1.18, 95% CI 1.01, 1.39, *P* = 0.04) and diabetes mellitus (OR 2.26, 95% CI 1.19, 4.30; *P* = 0.01). On multivariate analysis, in-hospital mortality was significantly decreased with appropriate empiric antibiotic therapy (OR 0.40, 95% CI 0.16, 1.00; *P* = 0.05), angiotensin-converting enzyme inhibitor therapy (OR 0.42, 95% CI 0.23, -0.77; *P* = 0.05) and antibiotic prophylaxis with Cefamandole (OR 0.46, 95% CI 0.19, 0.93; *P* = 0.03). On multivariate analysis, VAP was not associated with increased in-hospital mortality (OR 1.4. 95% CI 0.73, 2.50; *P* = 0.34). The Hosmer-Lemeshow goodness-of-fit test showed good calibration of the model (*P* = 0.87). The Nagelkerke and Cox/Snell *R*-squares were 0.29 and 0.22, respectively. Among the 162 (63% of the patients with POP) survivors, the median ICU length of stay was 19 (12 to 28) days in the VAP group versus 13 (9 to 22) days in the non-VAP group (*P* = 0.006).

## Discussion

This is the first study to compare VAP and non-VAP following CS with a special focus on microbiologic characteristics. In the present study, incidence of POP following CS was 3.5%, in agreement with previous studies, reporting incidence ranging from 2% to 10% [1-4,10]. However, previous studies confined their analysis to patients with VAP, which is not the only presentation of POP following CS, as illustrated by previous studies in which non-VAP represented almost 50% of all cases of POP following CS [6,11]. Few studies have compared VAP and non-VAP in the ICU [12-14] and only limited data are available on the antibiotic susceptibility of microorganisms isolated in this setting.

Few differences in terms of preoperative comorbidities were observed between the VAP and non-VAP groups, except for a larger proportion of NYHA class ≥ III patients and a more frequent history of congestive heart failure in the VAP group. Surgery was more difficult (longer operating time, longer cardiopulmonary bypass time, greater number of units of packed red blood cells transfused, etc.) in the VAP group than in the non-VAP group, explaining why these more severely ill patients (as illustrated by the higher severity score) were not extubated at the time of diagnosis of POP.

The microorganisms most commonly in this study isolated were Enterobacteriaceae (35%) and non-fermenting Gram-negative bacilli (22%) with no significant differences between the two groups of patients and according to the time of onset of POP. Studies evaluating POP following CS have reported similar results with a large proportion of Enterobacteriaceae and non-fermenting Gram-negative bacilli [1-4], but previous studies did not compare VAP and non-VAP. In the study by Esperatti *et al.* [12] comparing VAP and non-VAP, the authors also reported a high incidence of non-fermenting Gram-negative bacilli in the two groups of patients. Similarly, Rello *et al.* [14] did not find any differences in the microorganisms isolated between the groups of patients with VAP and non-VAP and the most commonly isolated microorganisms were Enterobacteriaceae (>20%) and non-fermenting Gram-negative bacilli (>20%).

Despite a high prevalence of early-onset POP in both groups, the most frequently prescribed antibiotic therapy was a broad-spectrum agent targeting non-fermenting Gram-negative bacilli (piperacillin/tazobactam in 70.4% of cases). This policy is justified by the high frequency of prior antibiotic use in the study population (21%) [11]. In studies evaluating POP following CS, we and others have reported a high incidence of non-fermenting Gram-negative bacilli, even in the first days following surgery [1,3,6].

Few studies have evaluated empiric antibiotic therapy in pneumonia in ICU. In the study by Rello *et al.* [14] assessing empiric antibiotic therapy in VAP and non-VAP, the most frequently prescribed antibiotic therapy included a broad-spectrum beta-lactam antibiotic targeting non-fermenting Gram-negative bacilli (34% of carbapenems and 22% of piperacillin/tazobactam) regardless of the type of pneumonia (VAP and non-VAP). The main difference was a higher proportion of carbapenems used in the study by Rello *et al.* (34%), whereas in our study only 13 patients (5%) received empiric antibiotic therapy including a carbapenem. This difference could be explained by the low and stable incidence of strains expressing extended-spectrum β-lactamase in our ICU (2.3%). In a study by Weber *et al.* [15] comparing VAP and non-VAP in ICU, a lower incidence of non-fermenting Gram-negative bacilli was observed in the non-VAP group compared to the VAP group (14% versus 32%, *P* <0.02). Based on the high incidence of resistant microorganisms, even in the non-VAP group (14%), these authors proposed the use of broad spectrum empiric antibiotic therapy in all cases of POP.

Antibiotic therapy prior to the onset of pneumonia is probably one of the most important factors associated with the isolation of resistant microorganisms, such as non-fermenting Gram-negative bacilli. Previous antibiotic treatments are known risk factors for *P. aeruginosa* infection in VAP [16-18] and in POP following CS [11].

In our study population, the time to onset of pneumonia (< or ≥5 days) and the type of POP (VAP or non-VAP) were not associated with an increased rate of isolation of non-fermenting Gram-negative bacilli. Similarly, Verhamme *et al.* [18] reported that the time to onset of VAP was not associated with isolation of multidrug-resistant pathogens (adjusted OR 1.1, 95% CI, 0.6, 2.2). Interestingly, prior antibiotic therapy (adjusted OR 8.2, 95% CI 2.8, 23.8) and antibiotic prophylaxis (adjusted OR 4.6, 95% CI 1.6, 13) were the strongest factors associated with the isolation of multidrug-resistant pathogens. A high selection pressure was observed in our study population, represented by preoperative antibiotic therapy in 21% of cases, and systematic antibiotic prophylaxis.

Despite a higher severity score in the ICU in the VAP group, in-hospital mortality was not significantly different between the two groups of patients. The absence of significant difference between the two groups of patients could be due to a lack of power. However, no difference was observed following univariate and multivariate analyses.

The mortality rate reported here (37%) is similar to those observed in previous studies evaluating POP following CS (between 24 and 46%) [1-4]. On univariate and in multivariate analysis, inappropriate antibiotic therapy was associated with poorer outcome with no significant differences between the 2 groups of patients. Many studies have also suggested that inappropriate antibiotic therapy for VAP is a risk factor for poorer outcome [14,19,20]. In our study, the prognosis was poor and similar between the two groups of patients. Previous studies comparing VAP and non-VAP [12-14] also did not report any difference in mortality between the two groups of patients.

Our study had a number of limitations. The data presented here correspond to routine management and were not specifically collected for this study. Moreover, only documented cases of POP were taken into account; which could lead to underestimation of the real POP rate, particularly in non-ventilated patients, who often present respiratory failure, therefore making the collection of respiratory-tract samples more difficult. Nevertheless, Fagon *et al.* [21] and Singh *et al.* [22] suggest that many non-infectious processes in postoperative patients are associated with lung infiltrates falsely attributed to pneumonia that could lead to overestimation of the incidence of pneumonia.

The conclusions based on this single-center study cannot be extrapolated to other institutions. Single-center studies are useful to demonstrate local ecological patterns, while larger studies may show regional or even global trends not apparent in smaller studies. All patients were included in this analysis, including those not mechanically ventilated at the time of the diagnosis of POP, whereas previous studies confined their analysis to patients with VAP [1-4]. We consider that extending the study population to non-VAP patients provides an interesting perspective for physicians in this field, as VAP is not the only clinical presentation of POP.

In the present study, only evaluating patients after CS, all patients were intubated and mechanically ventilated at the time of ICU admission and were extubated only when the standard extubation criteria were obtained. In contrast, in studies comparing VAP and non-VAP in general ICU [12-15], the reasons for ICU admission were variable and many patients were not intubated on admission, which can make it difficult to compare studies dealing with CS and general ICU.

## Conclusions

ICU patients with VAP are more severely ill than patients with non-VAP following CS. Nevertheless, mortality rates are similar between the two groups of patients. This study suggests that empiric antibiotic regimens in patients with pneumonia following CS should include at least a broad-spectrum antibiotic targeting non-fermenting Gram-negative bacilli, regardless of the type of pneumonia, and targeting *S. aureus* in VAP patients.

## Key messages

- Intensive care patients with VAP are more severely ill than non-VAP patients following CS
- Empiric antibiotic regimen should include at least a broad-spectrum antibiotic targeting non-fermenting Gram-negative bacilli, regardless of the type of pneumonia

## Additional file

Additional file 1:
**Appendix 1 Univariate analysis of risk factors for in-hospital mortality with** ***P*** **< 0.10.**

## Abbreviations

CFU: colony forming units
COPD: chronic obstructive pulmonary disease
CS: cardiac surgery
POP: postoperative pneumonia
VAP: ventilator-associated pneumonia.
