# Supplementary material for: Postoperative pneumonia following cardiac surgery in non-ventilated patients versus mechanically ventilated patients: is there any difference?
Source: Crit Care. 2015 Mar 11;19(1):116. doi: 10.1186/s13054-015-0845-5 (PMC4372228; doi:10.1186/s13054-015-0845-5)
Supplement: Additional file 1: — Appendix 1 Univariate analysis of risk factors for in-hospital mortality with P < 0.10. [file 13054_2015_845_MOESM1_ESM.doc]

| Appendix 1- Univariate analysis of risk factors for in-hospital mortality with *P*<0.10 | | | | |
| --- | --- | --- | --- | --- |
| Variable | Non-survivors | Survivors | Odds Ratio | *P-*value |
|  | (n =95) | (n =162) | (CI 95%) |
| Preoperative variables |  |  |  |  |
| Age (mean ± SD) (yr) | 71.5±10.7 | 67.3±12.6 | 1.03 (1.01-1.06) | 0.006 |
| History of congestive heart failure (n [%)]) | 31 (32.6) | 32 (19.8) | 1.97 (1.10-3.51) | 0.02 |
| Diabetes mellitus (n [%)]) | 36 (37.9) | 43 (26.5) | 1.69 (0.98-2.90) | 0.06 |
| Current smoking (n [%)])^a^ | 8 (8.4) | 33 (20.4) | 0.36 (0.16-0.82) | 0.01 |
| Creatinine clearance (mean ± SD) (mL/min) | 54.9±24.7 | 70.6±31.4 | 0.98 (0.97-0.99) | <0.0001 |
| Angiotensin-converting enzyme inhibitor (n [%)]) | 36 (37.9) | 92 (56.8) | 0.46 (0.28-0.78) | 0.003 |
| Statin (n [%)]) | 46 (48.4) | 104 (64.2) | 0.52 (0.31-0.88) | 0.013 |
| Intraoperative variables |  |  |  |  |
| Intraoperative number of units of packed red blood cells | 2.1±1.9 | 1.2±1.8 | 1.30 (1.13-1.50) | 0.0001 |
| Inotropic support at the end of surgery (n [%)]) | 59 (62.1) | 79 (48.8) | 1.72 (1.03-2.89) | 0.04 |
| Redo surgery (n [%)])^b^ | 19 (20) | 15 (9.3) | 2.45 (1.18-5.09) | 0.01 |
| Antibiotic prophylaxis with Cefamandole (n [%)]) | 74 (77.9) | 145 (89.5) | 0.41 (0.21-0.83) | 0.01 |
| Postoperative variables |  |  |  |  |
| Time to onset of pneumonia (days) | 4.3±1.9 | 3.7±1.7 | 1.23 (1.07-1.42) | 0.004 |
| POP with non-fermenting Gram-negative bacilli (n [%)]) | 37 (38.9) | 30 (18.5) | 2.81 (1.58-4.97) | 0.0003 |
| Appropriate empiric antimicrobial therapy (n [%)]) | 77 (81.1) | 151 (93.2) | 0.31 (0.14-0.69 | 0.003 |
| Ventilator-associated pneumonia (n [%)]) | 57 (60) | 80 (49.4) | 1.54 (0.92-2.57) | 0.10 |
| CI, confidence intervals; POP, postoperative pneumonia | | | | |
| ^a^Subject smoked an average of at least 5 cigarettes per day during the month before surgery. | | | | |
| ^b^Cardiac surgery requiring resternotomy. |  |  |  |  |
